# Late stage gastric cancer patients with extra gained HER2 positivity by dual block assessment may not show compromised efficacy to trastuzumab treatment

**DOI:** 10.18632/aging.102415

**Published:** 2019-11-17

**Authors:** Chen Xu, Yalan Liu, Dongxian Jiang, Xiaowen Ge, Jie Huang, Jieakesu Su, Xue Zhang, Shaohua Lu, Yuan Ji, Jun Hou, Tianshu Liu, Yingyong Hou

**Affiliations:** 1 Department of Pathology, Zhongshan Hospital, Fudan University, Shanghai, China; 2 Department of Oncology, Zhongshan Hospital, Fudan University, Shanghai, China

**Keywords:** gastric cancer, HER2, heterogeneity, trastuzumab, dual block

## Abstract

Dual block HER2 assessment can effectively increase the HER2 positive rate in resected specimens of gastric cancer (GC). The aim of this study is to explore whether GC patients with extra gained HER2 positivity by dual block assessment can benefit from trastuzumab therapy. Twenty-eight GC patients receiving gastrectomy prior to trastuzumab treatment were retrospectively analyzed. All the cases routinely accepted dual block HER2 assessment. The cases were divided into 2 cohorts based on HER2 status: cohort A with concordant HER2 results and cohort B with discordant HER2 results between the two blocks (cases with extra gained HER2 positivity). Response rate (RR), progress free survival (PFS) and overall survival (OS) were compared between the two cohorts. The results showed that no significant differences were found between the two cohorts in main clinicopathologic characteristics. No statistical difference was found in response rate (47.6% *vs* 57.1%) (*P*=1.0), either. The two cohorts did not demonstrate statistical differences in the PFS (10.5 months (95%CI 6.4-14.6) *vs* 8.0 months (95%CI 3.2-12.8), *P*=0.686) and the OS (23.3 months (95%CI 12.1-34.5) *vs* 20.0 months (95%CI 10.1-29.9), *P*=0.776). In conclusion, our study suggests that patients with extra gained HER2 positivity may not show compromised efficacy to trastuzumab treatment.

## INTRODUCTION

Gastric cancer is the fifth most common malignancy and the third common cause of cancer related death worldwide [1]. In China, it is the second and third most common cancer among male and female respectively, and the second leading cause of cancer death for both genders [2].

Early detection and treatment of GC is vital to acquire satisfying prognosis, however, many patients present with inoperable tumors during their first visit to hospitals in China, and for them, the prognosis is insufficient. Five-year survival rate of advanced GC patients is approximately 5% to 20%, and overall survival (OS) is approximately 10 months for those who receive conventional chemotherapy [3]. Recent years, molecular targeted therapy has been developed as a new strategy for late stage GC patients. The ToGA trial demonstrated that trastuzumab based chemotherapy could significantly improve the OS of late stage GC patients with human epidermal growth receptor 2 (HER2) positive tumors [4]. Since then, HER2 has become a vital predictive biomarker to select eligible patients for the targeted therapy in GC.

HER2 characteristics of GC have been extensively studied since the ToGA trial. Compared with breast cancer (BC), heterogeneity is an important feature in GC. Studies have shown that 30% to 79.3% of HER2 positive GCs show heterogeneity [5–8]. For this reason, HER2 assessment in GC is tougher than that in BC and false negative results may be frequently got [9, 10].

To cope with the adverse impact of HER2 heterogeneity on HER2 evaluation, we are the first to propose that dual block HER2 assessment (conducting HER2 assessment on two tumor-containing blocks) in resected specimens of GC is a practical way to increase HER2 positive rate [11, 12]. And since then, dual block HER2 assessment is routinely performed on resected specimens of GC in the Department of Pathology, Zhongshan Hospital. However, whether or not the patients with extra gained HER2 positivity by dual block assessment can benefit from the trastuzumab treatment is still to be elucidated.

Therefore, in the current cohort study, patients who accepted gastrectomy prior to trastuzumab treatment were retrospectively collected. Trastuzumab efficacy was compared between the two cohorts with concordant HER2 results or discordant HER2 results (extra gained HER2 positivity) between the two blocks. The purpose of this study is to explore if the patients with extra gained HER2 positivity are eligible candidates for the molecular targeted therapy.

## RESULTS

### Patient characteristics

Patient characteristics were shown in Table 1. Among the 28 eligible patients, there were 21 male and 7 female (male to female ratio 3:1). The median age was 62 years old (range 30–79). The mean age was 59.9 years old. With regard to tumor location, tumors of 5 cases were located in the oesophagogastric junction (OGJ), while the tumors of the remaining 23 cases were located in the stomach. As to Lauren classification, 18 cases were intestinal type, 5 were diffuse type, and the remaining 5 were mixed type. Thirteen cases showed moderate differentiation, and 17 patients demonstrated poorly differentiation.

**Table 1:** Patient characteristics.

	**Total, n(%)**	**Cohort A, n (%)**	**Cohort B, n (%)**	***P* value**
Gender				*P*=1.0
Male	21 (75.0)	16 (76.2)	5 (71.4)	
Female	7 (25.0)	5 (23.8)	2 (28.6)	
Age, median (range)	62.0 (30.0-79.0)			*P*=0.418
<60	12 (42.9)	8 (38.1)	4 (57.1)	
≥60	16 (57.1)	13 (61.9)	3 (42.9)	
Location				*P*=0.574
OGJ	5 (17.9)	3 (14.3)	2 (28.6)	
Stomach	23 (82.1)	18 (85.7)	5 (71.4)	
Lauren				*P*=0.393
Intestinal	18 (64.3)	15 (71.4)	3 (42.9)	
Diffuse	5 (17.9)	3 (14.3)	2 (28.6)	
Mixed	5 (17.9)	3 (14.3)	2 (28.6)	
Differentation				*P*=0.398
Moderate	13 (46.4)	11 (52.4)	2 (28.6)	
Poorly	17 (60.7)	10 (47.7)	5 (71.4)	
Stage after surgery				*P*=0.173
IIA	2 (7.1)	2 (9.6)	0 (0.0)	
IIB	4 (14.3)	3 (14.3)	1 (12.5)	
IIIA	5 (17.9)	5 (23.8)	0 (0.0)	
IIIB	4 (14.3)	1 (4.8)	3 (37.5)	
IIIC	4 (14.3)	3 (14.3)	1 (12.5)	
IV	10 (35.7)	7 (33.3)	3 (37.5)	
Median cycles of adjuvant chemotherapy, median (range)	4.0 (0-8)	5.5 (0-8)	2.5 (0-4)	*P*=0.131
Median cycles of palliative chemotherapy, median (range)	9.5 (2-34)	8 (2-34)	10 (5-20)	*P*=1.0
Median cycles of transtzumab, median (range)	8 (1-34)	8 (1-34)	6 (4-20)	*P*=1.0
Start of trastuzumab administration				*P*=0.072
First line	20 (71.4)	17 (81.0)	3 (42.9)	
Second line	7 (25.0)	3 (14.3)	4 (57.1)	
Third line	1 (3.6)	1 (4.8)	0 (0.0)	
Trastuzumab administration				*P*=0.674
Within line	10 (35.7)	7 (33.3)	3 (42.9)	
Cross line	18 (64.3)	14 (66.7)	4 (57.1)	
Response				*P*=1.0
CR/PR	14 (50.0)	10 (47.6)	4 (57.1)	
SD/PD	14 (50.0)	11 (52.4)	3 (42.9)	
Chemotherapy before surgery				*P*=0.551
No	25 (89.3)	18 (85.7)	7 (100.0)	
Yes	3 (10.7)	3 (14.3)	0 (0.0)	
Number of metastasis				*P*=0.633
<3	20 (71.4)	14 (66.7)	6 (4-20)	
≥3	8 ((28.6)	7 (33.3)	1 (14.3)	
mPFS (95%CI, months)	10.5 (5.5-15.5)	10.5 (6.4-14.6)	8.0 (3.2-12.8)	*P*=0.689
mOS(95%CI, months)	24.6 (17.3-31.9)	23.3 (12.1-34.5)	20.0 (10.1-29.9)	*P*=0.776

Abbreviations: OGJ: oesophagogastric junction; CR: complete response; PR: partial response; SD: stable disease; PD: progressive disease; mPFS: median progression free survival; mOS: median overall survival.

Trastuzumab was used as the first line treatment in 20 patients. Eighteen patients were given further trastuzumab administration beyond first line treatment progression. The total response rate was 50.0%. The mPFS of total patients was 10.5 months (range 5.5–15.5 months). And the mOS in total patients was 24.6 months (range 17.3–31.9 months).

### HER2 status

Among the 28 patients, 21 patients demonstrated concordant HER2 results between both blocks. These patients were enrolled into cohort A. Among them, 15 patients showed HER2 IHC 3+ in both blocks, and 6 patients showed HER2 IHC 2+/FISH+ in both blocks.

In the remaining 7 patients, the two blocks exhibited discordant HER2 results, and these patients were enrolled in cohort B (patients with extra gained HER2 positivity by dual block assessment). In 5 of the 7 patients, the first block was HER2 3+ and the second block was HER2 negative (HER2 2+/FISH- or HER2 0/1+). In the remaining 2 patients, the first block was HER2 2+/FISH+, and the second block was HER2 negative.

**Figure 1:**
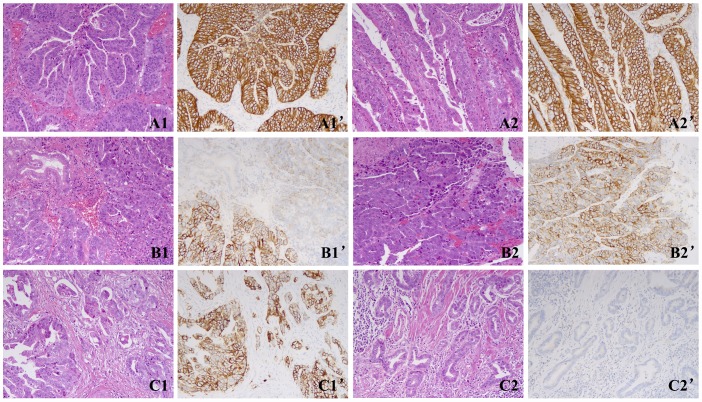
**Representative images of the two cohorts.** (**A**) A case of cohort A showed concordant HER2 results between the two blocks with homogeneous HER2 3+ staining. **A1**, **A1’**, Block 1; **A2**, **A2’**, Block 2. (**B**) A case of cohort A showed concordant HER2 results between the two blocks with heterogeneous HER2 3+ staining. **B1**, **B1’**, Block 1; **B2**, **B2’**, Block 2. (**C**) A case of cohort B showed discordant HER2 results between both blocks with 3+ in block 1 and 0 in block 2. **C1**, **C1’**, Block 1; **C2**, **C2’**, Block 2. Magnification ×200.

HER2 status was shown in Table 2 and Figure 1.

**Table 2:** HER2 status of both blocks in the two cohorts.

	**Block1**	**Block2**	**Case number**
Cohort A			
	3	3	15
	2	2	6
Cohort B			
	3	2	1
	3	1	1
	3	0	3
	2	1	1
	2	0	1

### Comparison of patients` characteristics between the two cohorts

The two cohorts demonstrated no statistical differences in gender (*P*=1.0), age (*P*=0.418), tumor location (*P*=0.574), Lauren classification (*P*=0.393), tumor differentiation (P=0.398), pTNM stage (according to the eighth edition of the Union for International Cancer Control (UICC) guidelines) (*P*=0.173), and number of metastasis (*P*=0.721) (Table 1).

With regard to the treatment profiles, the two cohorts showed no significant differences in the median cycles of ajuvant chemotherapy (*P*=0.131), palliative chemotherapy (*P*=1.0) and the trastuzumab administration (*P*=1.0) (Table 1).

### Comparison of trastuzumab response and survival analysis

**Figure 2:**
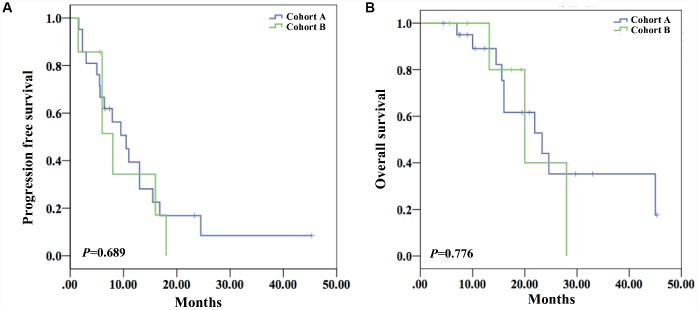
**Survival analysis of both cohorts.** (**A**) The mPFS of the two cohorts showed no statistical difference (10.5 months (95%CI 6.4-14.6) *vs* 8.0 months (95%CI 3.2-12.8)) (*P*=0.689). (**B**) The mOS of the two cohorts did not show statistical difference (23.3 months (95%CI 12.1-34.5) *vs* 20.0 months (95%CI 10.1-29.9)) (*P*=0.690).

The response rate of cohort A (47.6%) was slightly lower than cohort B (57.1%) with no statistical difference (*P*=1.0) (Table 1). With regard to survival analysis, the mPFS of cohort A was 10.5 months (95%CI 6.4-14.6), visually longer than that of cohort B (8.0 months (95%CI 3.2-12.8)) with no significant difference (*P*=0.689) (Figure 2). Similarly, mOS of cohort A (23.3 months (95%CI 12.1–34.5)) was slightly longer than that of cohort B (20.0 months (95%CI 10.1-29.9) with no statistical difference (*P*=0.690) (Figure 2).

## DISCUSSION

HER2 evaluation of GC can be challenging for the protein`s affinity of heterogeneous expression [7, 8, 10]. Because of the heterogeneity, false negative results may be got in HER2 assessment [9]. To cope with the heterogeneity and obtain more reliable HER2 results, we were the first to propose dual block assessment in resected specimens of GC [11]. Our previous studies indicated that it was a simple and practical way to increase HER2 positive rate [12]. For those with discordant HER2 results between the two blocks (one is HER2 positive and the other is not), there are possibilities to get false negative results with routine single block assessment. However, if the extra gained HER2 positive patients by the method could not benefit from trastuzumab treatment, simply increasing HER2 positivity is of limited clinical significance.

The current study showed that the RR, mPFS, mOS of both cohorts did not demonstrate statistical difference, although visually, mPFS and mOS were slightly shorter in the cohort B with extra gained HER2 positivity. This visual difference in the mPFS and mOS may be caused by the bias of the clinicopathologic features between the two cohorts. Although without statistical significance, more cases in cohort B were with later stage tumors than those in cohort A (IIIB-IV tumor proportion: 87.5% *vs* 52.4%), and more patients in cohort B started trastuzumab as the second line treatment. We mainly attribute such bias to small sample size. These result indicated that patients with extra gained HER2 positivity by dual block assessment may not show inferior treatment outcome to trastuzumab treatment. And these patients may also be eligible for the targeted treatment.

Because of the small sample size, the statistical power of this study was limited. The power was 17.6% for the mPFS and 5.19% for the mOS. To further confirm the findings, studies with larger sample size will be needed. Despite of the limited statistical power, the findings of this study are still of clinical importance. In our former studies, dual block assessment has been proved to effectively increase HER2 positive rate in GC. Yet little was known about how to deal with the extra gained HER2 positve patients with discordant HER2 results between the two blocks. To our knowledge, this is the first study to explore the clinical value of dual block HER2 assessment and it provides direct evidence that the late stage GC patients with extra gained HER2 positivity by dual block assessment may also benefit from trastuzumab treatment. Oncologists should pay more attention to the clinical significance of identifying such GC patients with heterogeneous HER2 positivity.

The impact of HER2 heterogeneity on trastuzumab efficacy has been studied in GC, but there are still controversies. Several studies showed that the heterogeneity may not adversely affect trastuzumab response. Xu et al. found that heterogeneity alone did not affect trastuzumab efficacy [13]. Van Cutsem et al. further analyzed the data from ToGA, and found that variability in staining intensity did not affect the overall benefit of trastuzumab, although the benefit was numerically lower for patients with variable staining tumors [14]. These findings were consistent with the current study. However, there were also studies showing the opposite. Wakatsuki et al. found that intratumoral HER2 heterogeneity may have robust impact on trastuzumab efficacy resected specimens in patients with HER2 positive GC [15]. Findings of Yagi et al. demonstrated that intratumoral HER2 heterogeneity using biopsy specimens showed clinical significance on trastuzumab efficacy [16]. These two studies both supported HER2 heterogeneity as a negative predictor.

In breast cancer (BC), homogeneously HER2 over expression showed more benefit from trastuzumab treatment [17]. Nevertheless, predictive relationship between the genetic heterogeneity and trastuzumab response was not found in early stage breast cancers in the adjuvant setting [18]. In addition, BC and GC are different tumor types with significant discrepancies in clinicopathologic characteristics and biological behavior. HER2 targeted agents, such as trastuzumab, lapatinib, T-DM1, and Pertuzumab, have all been successfully developed in BC, while all these agents failed to show benefit in GC except for trastuzumab [19–21]. Therefore, it is reasonable that the impact of HER2 heterogeneity on trastuzumab efficacy in GC is likely different from BC. Besides, even in BC, the cutoff to define HER2 3+ was adjusted from 30% of the invasive tumor cells to 10% [22]. This change in the cutoff indicated that HER2 heterogeneously positive BC can also benefit from the targeted therapy. Therefore, it is clinically reasonable to include patients with heterogeneous HER2 positivity as the candidates for molecular targeted therapy in both GC and BC.

There are some limitations in this research. First of all, it is a retrospective study from a single institution. Secondly, sample size is relatively small. This is mainly because most GC patients who accepted trastuzumab therapy are inoperable late stage patients. The direct impact of small sample size is the limited statistical power. Therefore, independent studies with larger sample size are needed to confirm the finding of this research.

In conclusion, GC patients with extra gained HER2 positivity by dual block assessment may not show compromised efficacy of trastuzumab treatment. Considering the retrospective nature of this study and limited statistical power, this result would need to be confirmed in independent studies. The findings provided the evidence of the clinical value of dual block HER2 assessment. Oncologists should be aware of the clinical significance of identifying GC patients with heterogeneous HER2 positivity. We recommended that dual block assessment routinely performed in resected specimens in GC to obtain more eligible patients for the molecular targeted therapy. With more oncoming data, conclusions of the current study can be further verified.

## MATERIALS AND METHODS

### Patients and treatment

One hundred and seventy five patients with advanced or metastatic gastric cancer of histologically confirmed HER2 positivity were given trastuzumab based chemotherapy in Zhongshan hospital, Fudan University from February 2010 to March 2018. Among them, 28 patients accepted gastrectomy prior to trastuzumab treatment and their primary tumor samples were available. All these patients accepted palliative trastuzumab based chemotherapy after tumor recurrence or metastasis. The HER2 status of all the 28 patients was routinely evaluated by dual block assessment. The research protocols were approved by the ethics board of Zhongshan Hospital. Prior written informed consents were received from all patients.

All the patients received standard palliative treatment including trastuzumab with a dose of 6 mg/kg every 3 weeks after a first infusion of 8 mg/kg. There were no protocol-specified chemotherapy regimens.

### Specimen handling, HER2 immunohistochemistry (IHC) staining and fluorescence in situ hybridization (FISH)

The specimens were fixed in 10% buffered formalin within 30 minutes after excision. Samples were processed following routine procedures after fixation for 24 hours. Haematoxylin and eosin staining was routinely performed.

HER2 IHC staining was conducted on a BenchMark XT automated stainer (Ventana Medical Systems, Inc., Tucson, AZ) with an iView DAB Detection Kit (Ventana, Tucson, AZ) according to the procedures previously described [23].

HER2 FISH was performed with a Pathvysion HER2 DNA Probe Kit (Abbott Laboratories, Des Plaines, Illinois). All the procedures followed the manufactures` instructions.

### HER2 evaluation

After pathological evaluation, two primary tumor blocks were chosen for HER2 IHC assessment. Blocks were given priority in the selection with the following conditions: containing an intestinal-type component, demonstrating the lowest grade and rich in tumor cells.

HER2 IHC status was evaluated by 2 independent observers who were blinded with the aim of the study. A discussion panel involving the third observer was introduced for cases with discrepant HER2 scores. HER2 was scored following the established criteria for resected specimens of GC [24–26]. Briefly, no staining or less than 10% of the tumor cells with positive staining was scored 0; faintly or barely perceptible staining on only a part of cell membrane in ≥10% of tumor cells was scored 1+; weak to moderate complete, basolateral, or lateral membranous reactivity in ≥10% of tumor cells was scored 2+; strong complete, basolateral, or lateral membranous reactivity in ≥10% of tumor cells was scored 3+. A case was regarded HER2 IHC positive if either of the 2 blocks was scored 3+.

For HER2 2+ blocks, HER2 FISH was performed to confirm the HER2 status. A case was considered to be with HER2 amplification when a minimum of 20 cancer cell nuclei exhibited a ratio of HER2: CEP17 (centromeric probe 17) of ≥2, or when HER2 signal clusters were observed.

A case was considered HER2 positive if either of the 2 blocks showed HER2 3+ or 2+/FISH+. Cases in which both 2 blocks were HER2 positive were considered to be those with originally gained HER2 positivity (cohort A). On the other hand, cases in which only 1 block showed HER2 positivity were considered to be those with extra gained HER2 positivity by dual block assessment (cohort B).

### Patient follow up

Medical records were reviewed to get the follow up data. Response was evaluated by CT/MRI scanning every 8 weeks after the trasutzumab administration following the Response Evaluation Criteria in Solid Tumors (RECIST) (version1.1) or earlier when indications of treatment failure were present. Patients were considered on study until death or loss to follow-up. The last date of follow up was December 20^th^, 2018.

Progression free survival (PFS) was defined as the time from the starting date of trastuzumab administration to the date of tumor progression. Overall survival (OS) was defined as the time from the start of trastuzumab treatment to the death of the patient. Response rate (RR) was defined as the ratio of the number of the patients with complete response (CR) plus partial response (PR) to the number of total patients.

### Statistical analysis

Clinicopathological parameters were evaluated with Chi-squared tests, Fisher's exact tests, and Mann-Whitney U test. Kaplan–Meier curves were constructed for survival analysis. Variables associated with prognostic value were selected to evaluate by univariate and multivariate analysis using Cox proportional hazard regression models. Data were presented as an HR and a 95% CI. For each analysis, a two-sided *P*-value of <0.05 was considered statistically significant. No adjustments were made. All the analyses were conducted with the SPSS version 22.0 (SPSS, Chicago, IL, USA).
